# Sorbitol treatment extends lifespan and induces the osmotic stress response in *Caenorhabditis elegans*

**DOI:** 10.3389/fgene.2015.00316

**Published:** 2015-10-27

**Authors:** Devon Chandler-Brown, Haeri Choi, Shirley Park, Billie R. Ocampo, Shiwen Chen, Anna Le, George L. Sutphin, Lara S. Shamieh, Erica D. Smith, Matt Kaeberlein

**Affiliations:** ^1^Department of Pathology, University of WashingtonSeattle, WA, USA; ^2^Department of Biology, Regis UniversityDenver, CO, USA; ^3^Department of Cell and Molecular Biology, Northwestern UniversityChicago, IL, USA

**Keywords:** osmotic stress response, glycerol, longevity, dietary restriction, sorbitol

## Abstract

The response to osmotic stress is a highly conserved process for adapting to changing environmental conditions. Prior studies have shown that hyperosmolarity by addition of sorbitol to the growth medium is sufficient to increase both chronological and replicative lifespan in the budding yeast, *Saccharomyces cerevisiae*. Here we report a similar phenomenon in the nematode *Caenorhabditis elegans*. Addition of sorbitol to the nematode growth medium induces an adaptive osmotic response and increases *C. elegans* lifespan by about 35%. Lifespan extension from 5% sorbitol behaves similarly to dietary restriction in a variety of genetic backgrounds, increasing lifespan additively with mutation of *daf-2(e1370)* and independently of *daf-16(mu86), sir-2.1(ok434), aak-2(ok524)*, and *hif-1(ia04)*. Dietary restriction by bacterial deprivation or mutation of *eat-2(ad1113)* fails to further extend lifespan in the presence of 5% sorbitol. Two mutants with constitutive activation of the osmotic response, *osm-5(p813)* and *osm-7(n1515)*, were found to be long-lived, and lifespan extension from sorbitol required the glycerol biosynthetic enzymes GPDH-1 and GPDH-2. Taken together, these observations demonstrate that exposure to sorbitol at levels sufficient to induce an adaptive osmotic response extends lifespan in worms and define the osmotic stress response pathway as a longevity pathway conserved between yeast and nematodes.

## Introduction

Studies in model organisms have demonstrated that aging can be influenced by a combination of genetic and environmental factors (Fontana et al., 2010; Kaeberlein, 2010; Kenyon, 2010). Genome-scale screens in both yeast and nematodes, for example, have identified several hundred genes that modulate lifespan, and multiple single-gene mutations have been shown to increase lifespan in rodents (Smith et al., 2007a; Yanos et al., 2012). Recently, attention in the field has turned toward the identification of pharmacological interventions that promote longevity and extend healthspan. High-throughput screens for compounds that increase lifespan have been reported in *C. elegans* (Petrascheck et al., 2009), and the United States National Institute on Aging Interventions Testing Program has tested the effect of nearly 30 different chemical agents on lifespan in mice, several of which have extended lifespan in at least one sex (Miller et al., 2007, 2011; Nadon et al., 2008; Harrison et al., 2009).

The best characterized environmental intervention for slowing aging is dietary restriction (DR), which can be defined as a reduction in nutrient availability in the absence of malnutrition. DR has been shown to enhance longevity and healthspan in a variety of different organisms including yeast, nematodes, flies, mice, dogs, and rhesus monkeys (Kennedy et al., 2007; Omodei and Fontana, 2011; Colman et al., 2014). One mechanism by which DR is thought to modulate aging is by reducing signaling through the mechanistic target of rapamycin (mTOR) kinase (Kapahi et al., 2010; Johnson et al., 2013).

In addition to food, temperature and oxygen availability are two environmental parameters that have a large effect on lifespan in *C. elegans* (Pitt and Kaeberlein, 2015). Wild type (N2) animals age more rapidly at high temperatures and more slowly at low temperatures, at least between a range from about 10–26°C (Klass, 1977; Hosono et al., 1982; Van Voorhies and Ward, 1999). Higher oxygen levels are associated with reduced lifespan, while hypoxia (0.5% oxygen) is sufficient to extend lifespan when applied during adulthood (Adachi et al., 1998; Mehta et al., 2009; Leiser et al., 2013). The genetic and molecular mechanisms underlying these observations remain unknown.

The osmotic environment experienced by an organism also has a large impact on cellular physiology and has been previously implicated in aging in the budding yeast *Saccharomyces cerevisiae*. Hyperosmolarity by addition of sorbitol or other non-metabolizable sugars at a concentration of 1M was shown to increase replicative lifespan, which is defined as the number of daughter cells a mother cell is capable of producing (Kaeberlein et al., 2002). This lifespan extension is dependent on the sirtuin deacetylase Sir2. Hyperosmolarity also increases yeast chronological lifespan, defined by the length of time a mother cell can survive in a non-dividing, quiescent like state during stationary phase (Smith et al., 2007b; Murakami et al., 2008). In this case, however, the lifespan extension is independent of Sir2 and is thought to involve induction of stress response genes that promote resistance to oxidative and pH stress (Burtner et al., 2009).

Hyperosmolarity induces a well-characterized response involving changes in cell cycle progression, coordinated changes in mRNA transcription and translation, and increased synthesis and cellular retention of organic osmolytes such as glycerol and trehalose (Saito and Posas, 2012). In *C. elegans*, it has been shown that animals exposed to elevated levels of sodium chloride undergo an osmotic adaptation that involves accumulation of glycerol (Lamitina and Strange, 2005). For worms maintained on standard NGM agar (50 mM NaCl), addition of NaCl at levels above 200 mM results in a significant reduction in survival after 24 h; however, if worms have been pre-adapted to high levels of NaCl, the loss of viability is attenuated (Lamitina et al., 2004; Lamitina and Strange, 2005). This protective effect of osmotic adaptation is correlated with reduced body size and requires intracellular accumulation of glycerol (Lamitina et al., 2004; Lamitina and Strange, 2005).

Here we report that, similar to the case in yeast, addition of sorbitol to the nematode growth medium is also sufficient to extend lifespan in *C. elegans*. Maximal lifespan extension is achieved when the medium is supplemented with around 5% sorbitol (275 mM) and is associated with increased expression of glycerol-3-phosphate dehydrogenase, accumulation of glycerol in the animals, and osmotic adaptation-induced resistance to NaCl stress. Genetic studies with known longevity pathways suggest that addition of sorbitol to the growth medium extends lifespan in *C. elegans* by a mechanism that is partially overlapping with DR.

## Results

### Increasing the osmolarity of the growth medium with sorbitol increases nematode lifespan and stress resistance

Longevity analysis in *C. elegans* is typically performed by maintaining animals on the surface of a nutrient-agar medium (nematode growth medium, NGM) with a lawn of *E. coli* OP50 as the food source. NGM is composed of peptone, salts, and cholesterol. A simple and robust method of DR for *C. elegans* has been described, in which animals are cultured in the standard conditions until early adulthood, at which time the OP50 food source is removed for the remainder of life (Kaeberlein et al., 2006; Lee et al., 2006; Smith et al., 2008; Sutphin and Kaeberlein, 2008). This method is referred to as bacterial deprivation (BD).

**Figure 1:**
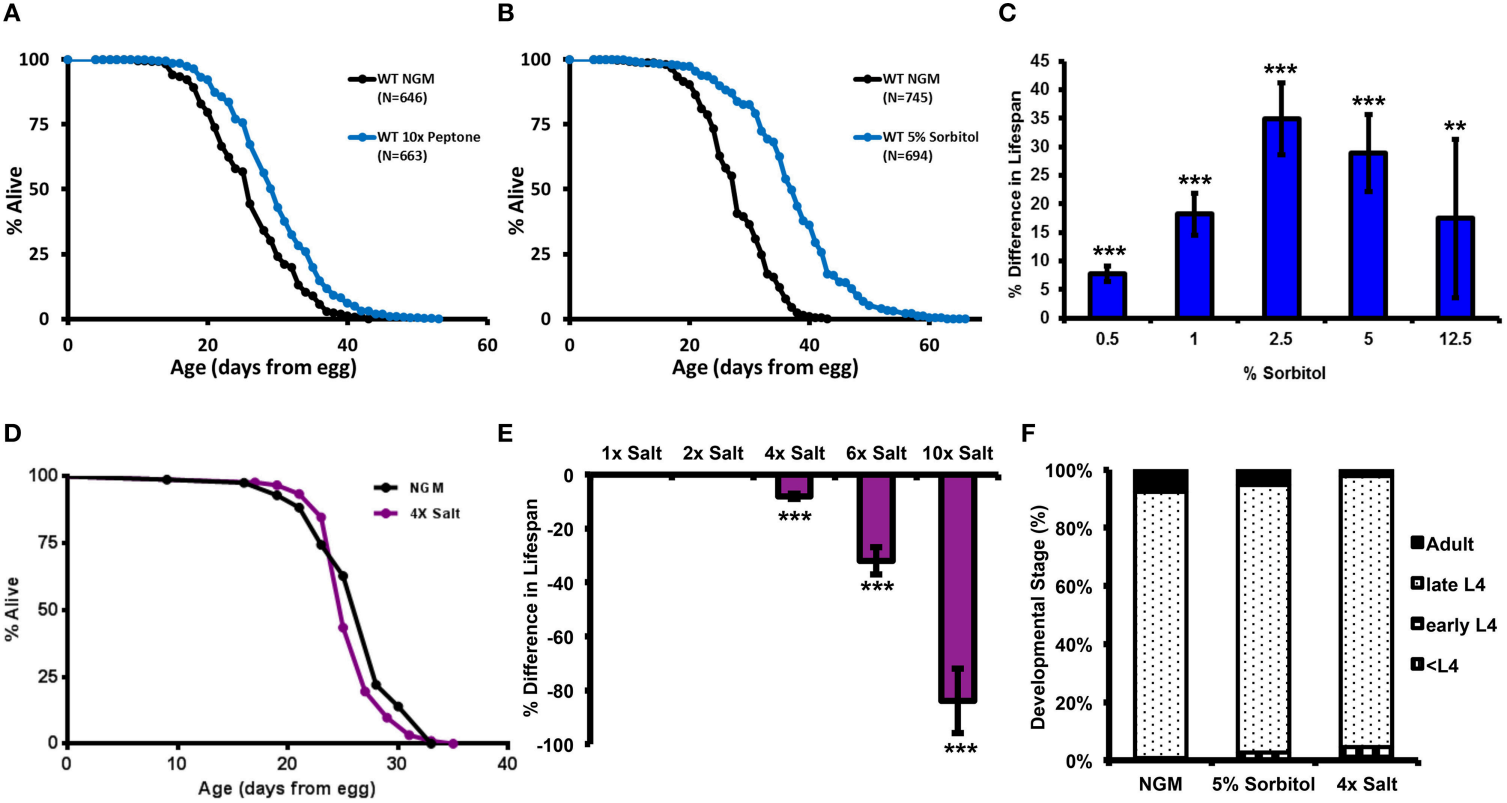
**Effect of Peptone, Sorbitol, and NaCl on lifespan in ***C. elegans*****. Lifespan is increased by addition of **(A)** a 10-fold higher concentration of peptone (2.5 g/L) or **(B)** 5% sorbitol to the nematode growth medium (NGM). **(C)** Effect of sorbitol on lifespan as a dose response. Addition of sorbitol to NGM at 0.5, 1, 2.5, 5, or 12.5% significantly extends lifespan (^**^p < 0.01, ^***^p < 0.005, *t*-test). **(D,E)** Addition of NaCl to NGM at 4, 6, or 10 times the normal concentration significantly decreases lifespan (^***^p < 0.001 in all cases, *t*-test). **(F)** Neither 5% sorbitol nor 4X NaCl significantly delayed development.

One unresolved question is whether BD animals require nutrients obtained from the peptone in NGM to achieve extreme longevity. In order to address this question, we performed a series of lifespan experiments in which the amount of peptone was varied both higher and lower than the standard concentration of 2.5 g/L. Relative to animals maintained on standard NGM, removing the peptone from the NGM had no detectable effect on the ability of BD to increase lifespan (Supplementary Figure 1A). While this does not rule out the possibility that BD animals are able to utilize components of the peptone as a nutrient source, it does demonstrate that peptone is not necessary for lifespan extension from BD. Unexpectedly, during this analysis we also observed that animals fed a normal diet and maintained on media supplemented with a 10-fold greater level of peptone (25 g/L) lived significantly longer than animals maintained on standard NGM (Figure 1A).

We hypothesized that 10X peptone might induce an osmotic adaptive response in *C. elegans* and that this could be related to lifespan extension. We therefore tested whether addition of the osmolytes NaCl or sorbitol could also promote longevity. NaCl is known to induce an osmotic adaptive response in *C. elegans* (Lamitina et al., 2004; Lamitina and Strange, 2005). Sorbitol is an acyclic polyol that is commonly used as an osmotic stabilizer, but has not been previously examined for effects on survival and adaptation to osmotic stress in *C. elegans*. Similar to the effect of 10X peptone, addition of 5% sorbitol (275 mM) to the NGM resulted in a significant lifespan extension (Figure 1B). The response to sorbitol appeared to be dose-dependent, with 0.5% sorbitol (27 mM) and 12.5% sorbitol (686 mM) increasing lifespan to a lesser extent than 2.5 or 5% sorbitol (Figure 1C, Table S2). In contrast to sorbitol, we failed to detect lifespan extension from NaCl at 200 mM (4X), and concentrations above this (300 mM; 6X or 500 mM; 10X NaCl) significantly reduced mean lifespan (Figures 1D,E, Table S2). Neither 5% sorbitol nor 4X NaCl resulted in a significant delay in development (Figure 1F).

**Figure 2:**
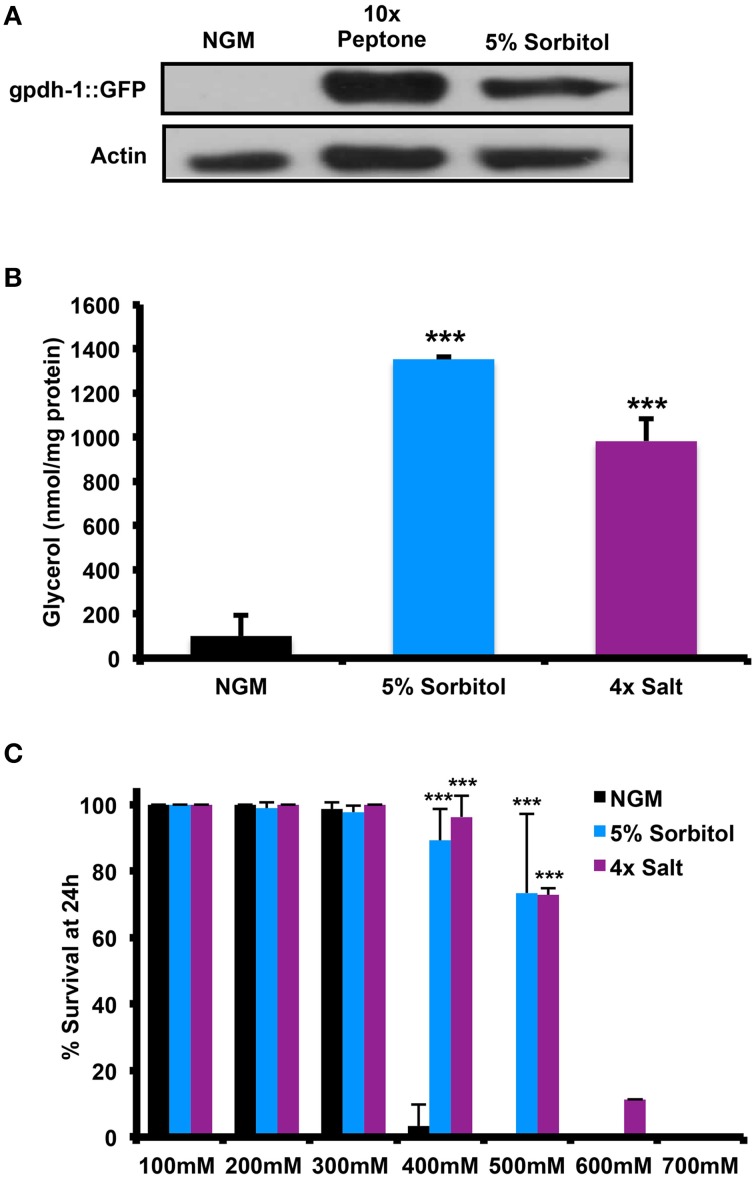
**Effect of Sorbitol on the accumulation of glycerol and resistance to high salt stress. (A)** Animals grown in medium containing 10X peptone or 5% sorbitol induce expression of the glycerol-3-phosophate dehydrogenase reporter pgdh-1::GFP, as determined by immunoblot analysis for GFP. **(B)** Whole animal glycerol levels were elevated in wild-type (N2) worms were grown on 5% sorbitol or 4X NaCl relative to controls (^***^p < 0.05, *t*-test). **(C)** Either 5% sorbitol or 4X NaCl is sufficient to confer resistance to higher levels of NaCl through osmotic pre-adaptation (^***^p < 0.05, *t*-test).

Since neither peptone nor sorbitol has been previously studied with respect to the adaptive osmotic response, we wished to determine whether this response is induced under lifespan-extending conditions. One feature of the adaptive osmotic response to NaCl in *C. elegans* is accumulation of intracellular glycerol through upregulation of the glycerol-3-phosphate dehydrogenase gene *gpdh-1* (Lamitina et al., 2004; Lamitina and Strange, 2005). Similar to treatment with NaCl, we find that addition of either 5% sorbitol or 10X peptone was induces expression of *gpdh-1*, as determined by accumulation of GFP driven by the *gpdh-1* promoter in a P_gpdh-1_::GFP transgenic strain (Figure 2A). 5% sorbitol also increased accumulation of glycerol within the animals to an extent comparable to 4X NaCl (Figure 2B). A second hallmark of the adaptive osmotic response is enhanced resistance to high levels of NaCl following pre-adaptation at a lower level of NaCl (Lamitina et al., 2004; Lamitina and Strange, 2005). Pre-treatment with either 5% sorbitol or 4X NaCl led to enhanced resistance to high levels of NaCl, with 5% sorbitol nearly as effective as pre-treatment with NaCl (Figure 2C).

**Figure 3:**
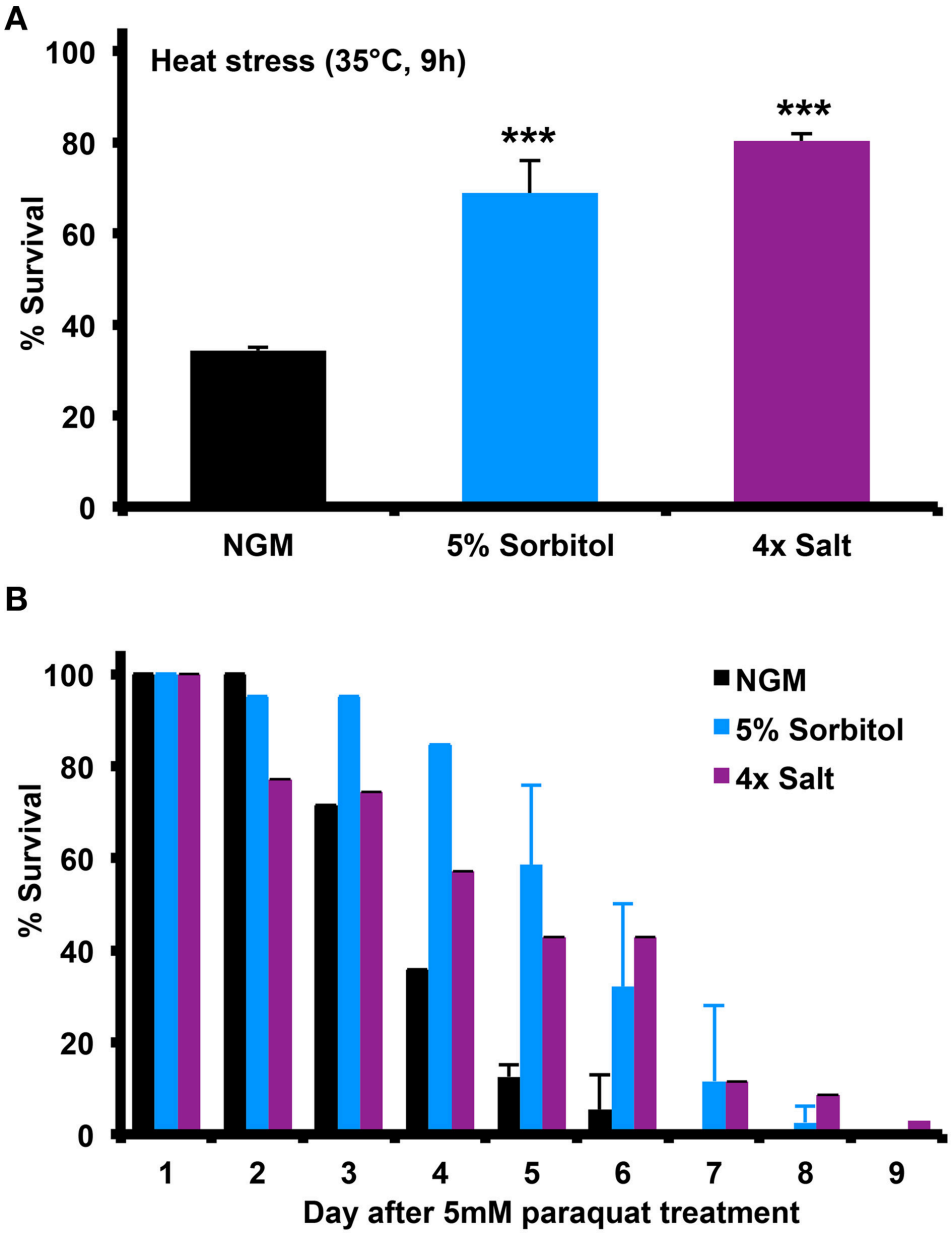
**Increasing the osmolarity of the growth medium with sorbitol increases nematode stress resistance**. Addition of 5% sorbitol or 4X NaCl to NGM both significantly enhanced resistance to **(A)** heat shock stress, as assessed by survival following 9 h at 35°C (^***^p < 0.05, *t*-test), or **(B)** survival during exposure to the superoxide-generating agent paraquat (5 mM).

Many long-lived mutants in *C. elegans* show enhanced resistance to different forms of stress, including heat shock and oxidative stress. To determine whether induction of the osmotic stress response can induce similar stress resistance, we assessed the ability of animals to survive heat shock at 35°C or in the presence of the mitochondrial superoxide generator paraquat. Relative to NGM, 5% sorbitol and 4X NaCl both significantly enhanced resistance to both forms of stress (Figures 3A,B).

### Sorbitol promotes longevity by a mechanism distinct from insulin-like signaling, sir-2.1, AMP kinase, and the hypoxic response

**Figure 4:**
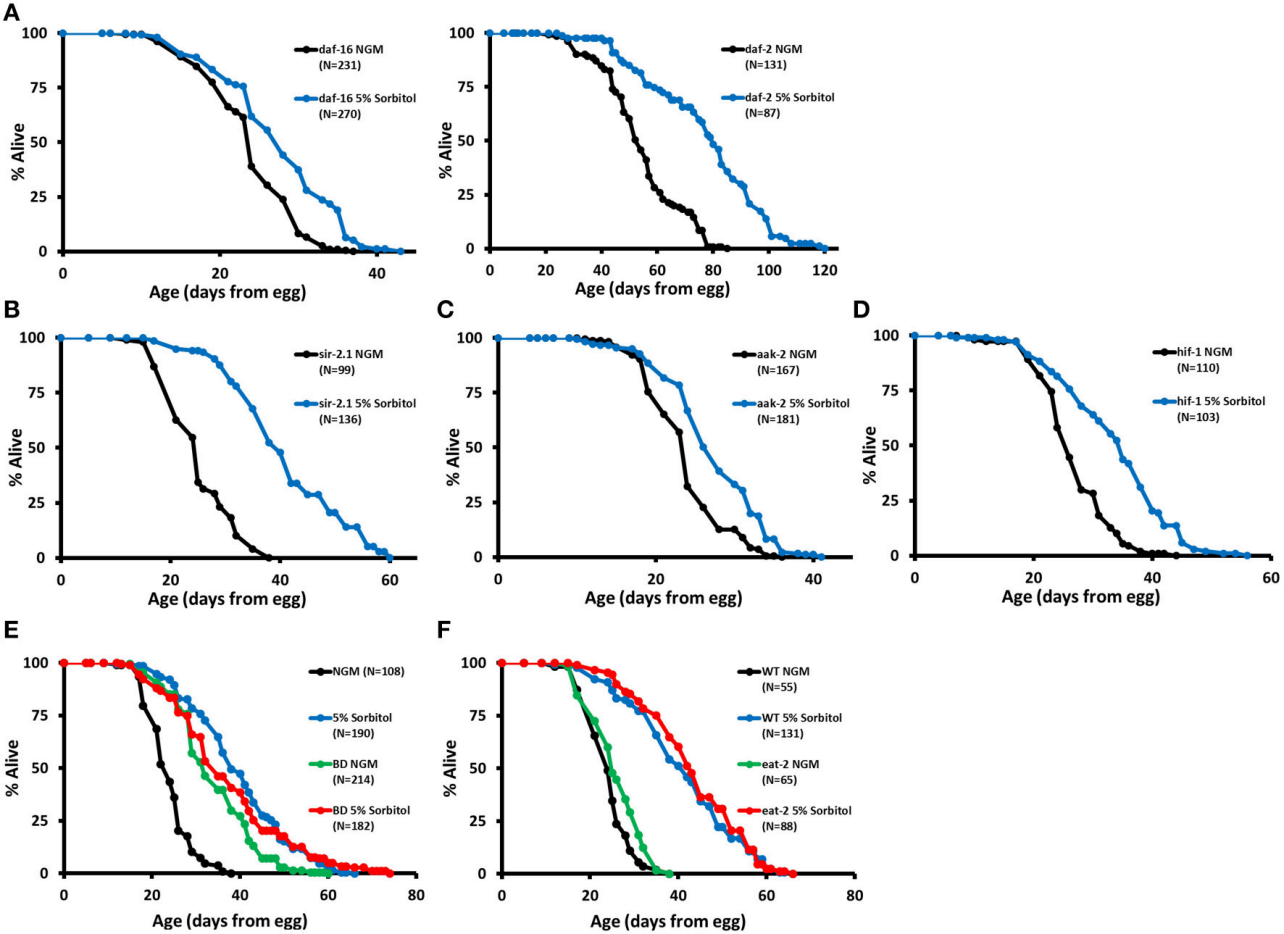
**Sorbitol promotes longevity by a mechanism distinct from insulin-like signaling, sir-2.1, AMP kinase, and the hypoxic response**. **(A)** Addition of 5% sorbitol to the NGM extends the lifespan of both *daf-16(mu86)* and *daf-2(e1370)* mutants (*p* < 0.001, *t*-test). Addition of 5% sorbitol to the NGM is also sufficient to extend lifespan in **(B)** *sir-2.1(ok434)* null animals, **(C)** *aak-2(ok524)* null animals, and **(D)** *hif-1(ia04)* null animals (*p* < 0.001 in each case, *t*-test). **(E)** Dietary restriction by bacterial deprivation (BD) or **(F)** mutation of *eat-2* fails to further extend the lifespan of animals grown on NGM supplemented with 5% sorbitol (*p* = 0.21, *t*-test).

Insulin-like signaling is a key regulator of longevity in *C. elegans*, and several mutations that reduce signaling through this pathway have been shown to increase lifespan, including mutation of the insulin-like receptor *daf-2* (Kenyon, 2010). Lifespan extension from reduced insulin-like signaling results from nuclear localization and enhanced activity of the FOXO-family transcription factor DAF-16 (Lin et al., 1997; Ogg et al., 1997). Hence, a defining characteristic of longevity mutations acting in this pathway is that lifespan extension is fully suppressed by a null mutation in *daf-16*. In contrast to mutations that extend lifespan by reducing insulin-like signaling, 5% sorbitol significantly extended the lifespan of *daf-16(mu86)* null animals (Figure 4A) and further extended the lifespan of long-lived *daf-2(e1370)* animals (Figure 4A).

Sirtuin deacetylases have been implicated in longevity control in both yeast and *C. elegans*. Overexpression of Sir2 in yeast (Kaeberlein et al., 1999) or SIR-2.1 in nematodes (Tissenbaum and Guarente, 2001) leads to increased lifespan in each organism, although the effect of SIR-2.1 overexpression in worms is somewhat controversial (Burnett et al., 2011; Viswanathan and Guarente, 2011). Addition of sorbitol to the growth medium requires Sir2 for replicative lifespan extension in yeast (Kaeberlein et al., 2002; Burtner et al., 2009). In contrast to this, the *sir-2.1(ok434)* null allele did not prevent lifespan extension from 5% sorbitol in *C. elegans* (Figure 4B).

AMP activated protein kinase has also been shown to promote longevity in *C. elegans*. Overexpression of the AMP catalytic subunit (AAK-2) is sufficient to increase lifespan, and *aak-2* is required for lifespan extension from deletion of the S6 kinase homolog gene *rsks-1* and at least one form of DR (Apfeld et al., 2004; Curtis et al., 2006; Greer et al., 2007; Selman et al., 2009; Harel et al., 2015). Lifespan extension from sorbitol is independent of *aak-2*, as evidenced by the significant lifespan extension in *aak-2(ok524)* animals on NGM supplemented with 5% sorbitol (Figure 4C).

We also examined whether 5% sorbitol might be acting to promote longevity through activation of the hypoxic response transcription factor HIF-1, which has been shown to act as both a positive and negative modulator of longevity in *C. elegans* (Kaeberlein and Kapahi, 2009; Leiser and Kaeberlein, 2010). HIF-1 is negatively regulated by proteasomal degradation under normoxic conditions. Stabilization of HIF-1 through deletion of the *vhl-1* E3 ligase or transgenic expression of a non-degradable allele is sufficient to increase lifespan (Mehta et al., 2009; Müller et al., 2009; Zhang et al., 2009; Leiser et al., 2011). Unlike deletion of *vhl-1*, addition of 5% sorbitol significantly increased the lifespan of *hif-1(ia4)* mutants (Figure 4D).

### Dietary restriction fails to further extend lifespan in combination with 5% sorbitol

Several methods for extending lifespan in *C. elegans* through DR have been described (Harel et al., 2015). Most methods of DR share several features, including: additive lifespan extension when combined with mutation of *daf-2(e1370)* and lifespan extension in animals mutated *for daf-16(mu86), hif-1(ia04), sir-2.1(ok434), or aak-2(ok524)*. Since this pattern of interaction matched that of 5% sorbitol, we hypothesized that 5% sorbitol may be acting as a DR mimetic. Consistent with this idea, two different methods of DR, BD and the *eat-2(ad1113)* mutation fail to further extend the lifespan of animals in the presence of 5% sorbitol. (Figures 4E,F).

### The osmotic response pathway is associated with lifespan extension

**Figure 5:**
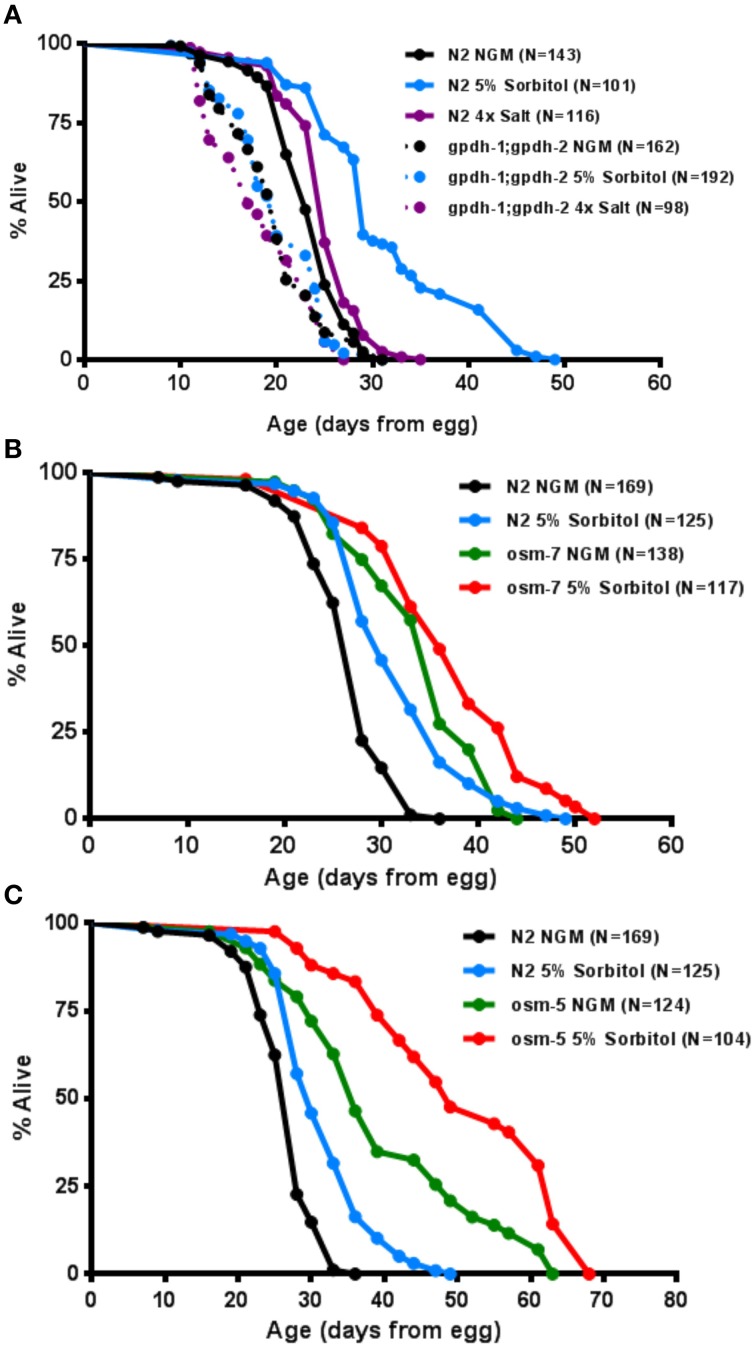
**The osmotic response pathway is associated with lifespan extension**. **(A)** Genetic ablation of induced glycerol biosynthesis in the *gpdh-1(ok1558);gpdh-2(kb33)* double mutants prevents lifespan extension from 5% sorbitol. **(B)** Two mutations with constitutive activation of the osmotic stress response, *osm-5(p813)* and *osm-7(n1515)*, are long-lived under control conditions (*p* < 0.001, *t*-test).

To more directly assess the importance of the adaptive osmotic response in lifespan extension from sorbitol, we asked whether induction of the glycerol biosynthetic machinery is necessary for enhanced longevity. The *C. elegans* genome contains two glycerol-3-phosphate dehydrogenase genes, *gpdh-1* and *gpdh-2*, which encode the rate-limiting enzyme for glycerol biosynthesis (Lamitina et al., 2006). These genes are induced as part of the osmotic response and facilitate the accumulation of intracellular glycerol. Animals carrying mutations in both *gpdh-1(ok1558)* and *gpdh-2(kb33)* did not exhibit increased lifespan in the presence of 5% sorbitol (Figure 5A).

To determine whether induction of the osmotic stress response pathway might be sufficient to increase lifespan, we examined the effects of mutations in two different genes that are known to promote osmotic stress resistance in worms. Each of these mutants, *osm-5(p813)* and *osm-7(n1515*), showed a robust extension of lifespan (Figures 5B,C). Both mutants also had their lifespans further extended by 5% sorbitol. Taken together these data demonstrate that the glycerol-3-phosphate dehydrogenase genes, *gpdh-1* and *gpdh-2*, are necessary for lifespan extension from sorbitol and suggest that induction of the osmotic stress response is associated with extended lifespan under some conditions.

## Discussion

Aging is a complex process that is strongly influenced by both environmental and genetic factors. Of particular interest are those factors that modulate aging across evolutionarily divergent species. Here we have shown that sorbitol, which is known to increase lifespan and induce an osmotic response in the budding yeast, has similar effects in *C. elegans*. Our data suggest that the pro-longevity effect of sorbitol in worms may be mediated in part by a mechanism similar to DR, as well as through induction of the osmotic stress response pathway.

Although sorbitol can extend lifespan in both yeast and worms, it remains unclear whether the downstream mechanisms are shared. Unlike the case for yeast replicative lifespan (Kaeberlein et al., 2002), lifespan extension from sorbitol in *C. elegans* does not require the Sir2 homolog *sir-2.1*. Subsequent studies in yeast have indicated that deletion of Sir2 prevents replicative lifespan extension of most longevity interventions and mutants in a non-specific manner, however, and therefore failure to extend lifespan in cells lacking Sir2 cannot be interpreted as a direct mechanistic interaction (Kaeberlein et al., 2004; Delaney et al., 2011). Interestingly, deletion of the yeast homolog of *gpdh-1* and *gpdh*-2 prevents lifespan extension from sorbitol in yeast (Kaeberlein et al., 2002), similar to the case in worms, suggesting the possibility that activation of these enzymes may underlie the longevity effect in both cases.

Our data support the model that lifespan extension from sorbitol in worms is mechanistically similar to DR and mechanistically distinct from other longevity-enhancing mutations and interventions that act through insulin-like signaling, SIR-2.1, AMP kinase, or the hypoxic response. It should be noted, however, that these types of longevity epistasis results do not allow us to rule out the possibility that sorbitol acts partially through these other pathways. For example, the magnitude of lifespan extension from sorbitol appears to be reduced in animals lacking *aak-2* or *daf-16*, which could be interpreted to suggest that lifespan extension is mediated partially through activation of these factors. Likewise, the failure of sorbitol to further extend lifespan in two different *C. elegans* models of DR is consistent with, but not definitive proof, for the idea that sorbitol and DR act via similar downstream mechanisms. It should also be noted that in our study the *eat-2(ad1113)* mutation had only a modest effect on lifespan that did not reach significance at the *p* < 0.05 level. This may reflect a combination of the severe pumping defect of this mutant combined with the conditions used in our study, such as growth-arrested food. There is growing evidence that the magnitude, and even direction, of effect from DR is highly dependent upon both genetic and environmental factors in a variety of model organisms from yeast to monkeys (Liao et al., 2010; Swindell, 2012; Schleit et al., 2013; Colman et al., 2014), and it is therefore not surprising that the *eat-2(ad1113)* mutant shows a variable magnitude of response under different conditions. Nonetheless, the results with this mutant, as well as the BD form of DR both show a failure of DR to extend lifespan in animals treated with sorbitol.

One possible mechanism is that induction of the osmotic stress response may prevent or delay age-associated loss of protein homeostasis (Lamitina et al., 2006; Choe and Strange, 2008). This is consistent with our observation that addition of sorbitol enhances resistance to both heat shock and paraquat treatment. Production of small molecule metabolites may also contribute to lifespan extension in sorbitol-treated nematodes. Both trehalose and glycerol biosynthesis are upregulated in response to osmotic stress, and it has been reported that addition of trehalose to the NGM is sufficient to extend lifespan in *C. elegans* (Honda et al., 2010). In the case of exogenous trehalose, however, lifespan extension is mediated by the IIS pathway, suggesting that this is unlikely to be the only mechanism involved in lifespan extension from sorbitol (Honda et al., 2010).

A complication of studies of aging in *C. elegans* is that the bacterial food source can limit lifespan, influence the composition of the growth medium, and alter the manner in which the animals respond to changes in the environment (Garigan et al., 2002; Garsin et al., 2003). While we cannot completely rule out the possibility that sorbitol is influencing longevity through effects on the bacterial food source, we believe that this is unlikely, since our standard protocol is to use UV-arrested *E. coli* OP50 for longevity experiments (Sutphin and Kaeberlein, 2009). Thus, growth inhibition of the bacteria by sorbitol does not contribute to the effects we are reporting here. Addition of sorbitol also increases lifespan when live OP50 are used (Supplementary Figure 1B), further suggesting that the pro-longevity effects of this regimen are unlikely to be bacterially mediated.

At this time we are unable to explain the apparent differences in the effect that various osmolytes have on longevity. Although both peptone and sorbitol extend lifespan, none of the concentrations of NaCl that were tested significantly increased lifespan. Addition of sucrose at 305, 484, or 652 mM has also been reported to reduce 24-h survival of *C. elegans* (Lamitina and Strange, 2005). Although the effect of sucrose on lifespan has not been reported, it seems unlikely that lifespan extension would be observed if death after 24 h is significant. Importantly, we did not detect reduced 24-h survival from any of the concentrations of sorbitol tested. Thus, it may be that NaCl and sucrose have additional toxic effects that limit their ability to extend lifespan. Alternatively, it may be that sorbitol engages additional pro-longevity mechanisms that are not induced by NaCl and perhaps other osmotic stressors.

The response to osmotic stress is highly evolutionarily conserved, and it is of particular interest that this response, at least in the case of sorbitol, extends lifespan in both yeast and nematodes. This is akin to other environmental cues such as temperature, oxygen availability, and nutrient abundance, that also modulate aging across large phylogenetic distance (Pitt and Kaeberlein, 2015). We speculate that this reflects a fundamental relationship between stress resistance, development, and longevity. Under conditions that are favorable for growth (i.e., abundant nutrients, optimal temperature, salinity, and oxygen), organisms have evolved to develop and reproduce rapidly, perhaps at the expense of shortened lifespans. In contrast, where one or more of these environmental parameters is unfavorable, organisms induce stress response mechanisms that tend to be arrest or delay development and reproduction until the environmental conditions normalize. These same stress response mechanisms also tend to promote enhanced longevity, through a variety of potential mechanisms including enhanced proteostasis and protein turnover, altered metabolism and mitochondrial function, and elevated xenobiotic detoxification. Given the highly conserved and evolutionarily ancient nature of these key stress response pathways, it is tempting to speculate that their regulation by environment and their downstream effects on cellular and organismal longevity are also likely to be conserved in more complex animals, as appears to be the case for DR.

In this study, we have focused primarily on sorbitol, because it yields a robust and reproducible lifespan extension under our standard conditions. It will be of interest to further explore the physiological consequences of addition of sorbitol to the NGM vs. addition of NaCl. It will also be interesting to determine whether other methods for inducing the osmotic adaptive response, or specific target genes involved in this response, lengthen, shorten, or have no effect on lifespan in *C. elegans*. Defining these processes will provide important insight into the mechanisms of longevity control in *C. elegans* and may provide new targets for intervening directly in the aging process.

## Materials and methods

### Strains and growth conditions

*C. elegans* strain maintenance and manipulation were performed using standard methods, as previously described (Kaeberlein et al., 2006; Smith et al., 2008; Sutphin and Kaeberlein, 2008). Unless otherwise stated, animals were maintained on solid NGM [50 mM NaCl, 0.25% Bacto Peptone (BD Biosciences), 2% agar, 1 mM MgSO_4_, 1 mM CaCl_2_, 12.9 μM Cholesterol, 9.75 mM K_3_PO_4_ pH 6.0] supplemented with 50 μg/ml ampicillin with UV-arrested *E. coli* OP50 as the food source. Lifespan studies were performed according to our previously published protocol (Sutphin and Kaeberlein, 2009) and were carried out at 20°C. The longer absolute lifespan of N2 in this study is likely to reflect the use of growth arrested bacteria, as dividing *E. coli* are known to limit the lifespan of *C. elegans* (Garigan et al., 2002; Garsin et al., 2003). Statistical analysis and replication of lifespan experiments is provided in Table S1. Ruptured animals were not censored from lifespan experiments. Animals that foraged off the surface of the plate during the course of the experiment were not considered. Nematode strains used in this study are described in Table S2.

### Statistical analysis

*T-test* was used to generate *p*-values to determine statistical significance for lifespan assays. The mean lifespans, number of animals, number of replicate experiments, and *p-values* are provided in Table S1.

### Western blotting

Worms were synchronized by hypochlorite extraction of eggs and exposed to control or experimental conditions beginning on day 2 of adulthood. Protein was extracted 24 h later by 3 rounds of freeze-thaw cracking and dounce homogenization in a homogenization buffer [15 mM Hepes (pH 7.6), 10 mM KCl, 1.5 mM MgCl_2_, 0.1 mM EDTA, 0.5 mM EGTA 44 mM, 1 mM DTT, 2 mM NaVO_4_, 10 μg/ml Aprotinin, 10 μg/ml Leupeptin, 1x Phosphatase Inhibitor Cocktail 2 (Sigma)]. Samples were run on a 4–12% Bis-Tris gel in MOPS buffer and transferred to a PVDF membrane. Blots were blocked for 15 min in 5% nonfat dehydrated milk and probed for 1 h with either a mouse α-HIF-1 antibody in whole serum provided by Dana Miller (Budde and Roth, 2010) or mouse α-actin antibody MAB150 (Millipore) diluted 1:10000 in 1% milk. Santa Cruz Biotechnology secondary HRP-conjugated goat α-mouse IGG antibody (sc-2064) was diluted 1:5000 in 1% milk and incubated for 1 h. SuperSignal chemiluminescent reagents (Thermo Scientific) were used as HRP substrate according to the manufacturer's recommended procedure. Abcam ab69312 and Santa Cruz Biotechnology sc-9996 were used at a 1:2500 dilution for anti-GFP immunoblotting.

### Glycerol quantitation

Wild type animals were grown at 20°C on NGM and transferred at the L4 stage to control or hypertonic agar (5% sorbitol or 200 mM NaCl). Animals were maintained under these conditions (500 worms per condition) for 4 days and then harvested. Well-fed worms were rinsed off plates with M9 buffer, spun down, and then rinsed with fresh M9 twice and resuspended in 500 μl M9. 500 μl of the pellet was dropped by transfer pipette into liquid nitrogen. Frozen worms were ground to a fine powder with homogenizer cooled in nitrogen. The power was neutralized with 1N perchloric acid (PCA) to extract organic solutes and precipitate proteins. After centrifugation, the acid supernatant was neutralized with 10N KOH. Glycerol levels were measured with a commercially available UV-based glycerol assay kit (R-Biopharm Inc, Washington, MO USA). PCA precipitated pellets were solubilized with 0.1N NaOH, and protein content was measured with BCA assay Kit (Thermo Fisher Scientific Inc, Rockford, IL USA) and used for the normalization of glycerol levels.

### Conflict of interest statement

The authors declare that the research was conducted in the absence of any commercial or financial relationships that could be construed as a potential conflict of interest.
