# Supplementary material for: Sorbitol treatment extends lifespan and induces the osmotic stress response in Caenorhabditis elegans
Source: Front Genet. 2015 Oct 27;6:316. doi: 10.3389/fgene.2015.00316 (PMC4621483; doi:10.3389/fgene.2015.00316)
Supplement: Supplementary file 1 [file DataSheet1.DOCX]

Supplemental Materials for: **Lifespan extension from induction of the osmotic stress response in *Caenorhabditis elegans***

**Devon Chandler-Brown^1,a^, Haeri Choi^1,a^, Shirley Park^1^, Billie R. Ocampo^1^, Shiwen Chen^1^, Anna Le^1^, George L. Sutphin^1^, Lara S. Shamieh^2^, Erica D. Smith^3*^, Matt Kaeberlein^1*^**

^1^Department of Pathology, University of Washington, Seattle, WA 98195, USA.

^2^Department of Biology, Regis University, Denver, CO, 80221

^3^ Department of Cell and Molecular Biology, Northwestern University, Chicago, IL, 60611, USA


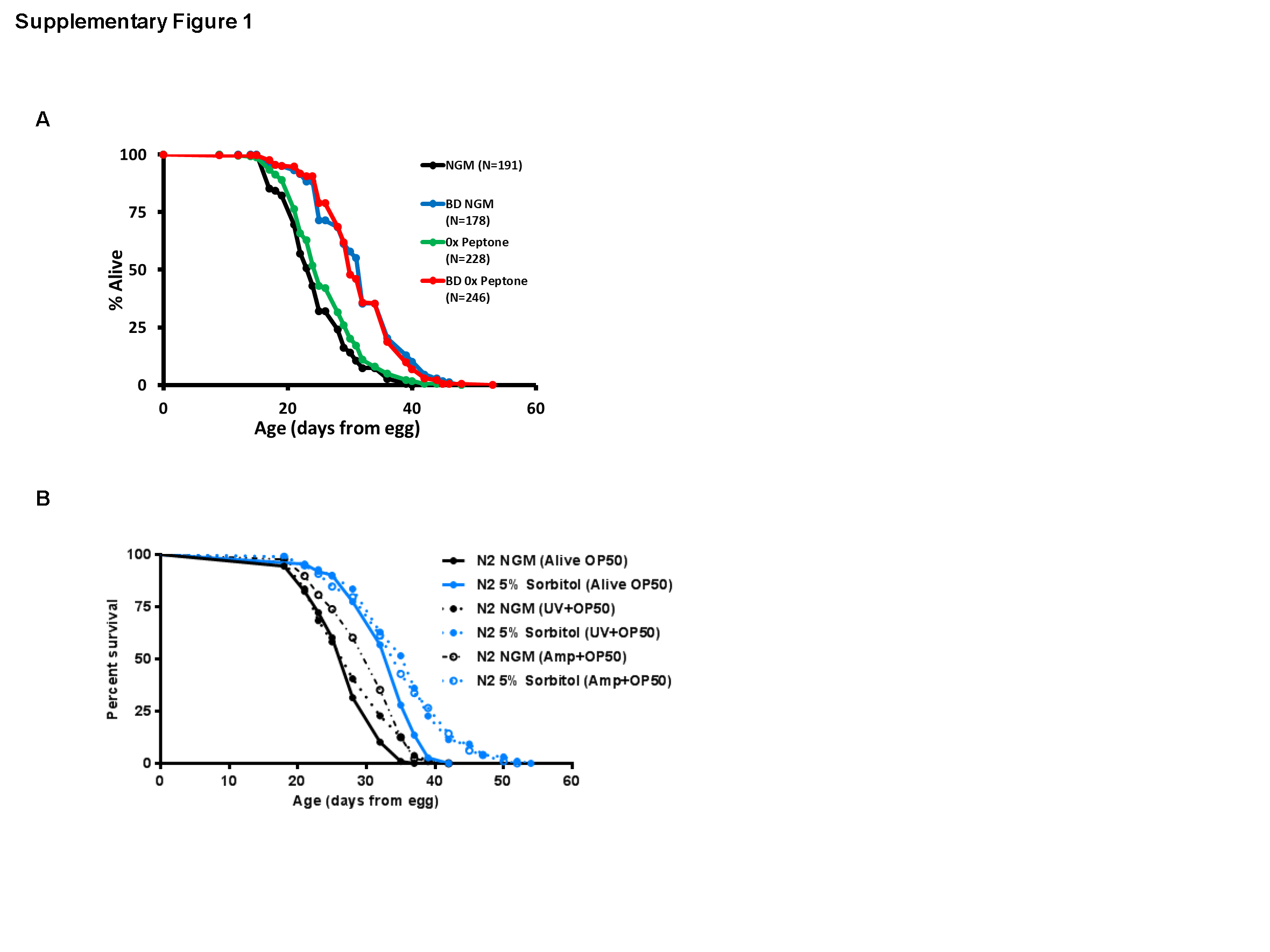


**Supplementary Figure 1. Peptone is required for lifespan extension from dietary restriction. (A)** Removal of the peptone from the nematode growth medium (NGM) does not significantly alter lifespan (*p = 0.79, t-test*), nor does it prevent lifespan extension by bacterial deprivation (BD) (*p < 0.001, t-test* for BD relative to control fed in both cases). **(B)** Survival curves of N2 wild-type *C. elegans* fed live *E. coli* OP50, UV-arrested *E. coli* or Ampicillin arrested *E. coli* on solid nematode growth medium (NGM, *p < 0.001, t-test* for sorbitol relative to control fed, in all cases).

**Table S1. Data from individual life span experiments.** All experiments were performed at 20°C on NGM with UV-arrested E. coli OP50 food and supplemented as indicated. BD refers to bacterial deprivation by removal of the bacterial food source during adulthood. *Condition Matched; **Genotype/Condition 2 Matched; ***Genotype/Condition 1 Matched

| **Strain** | **Condition 1** | **Condition 2** | **n** | **Mean** | **% Extension*** | | **% Extension**** | | **% Extension***** | **Median** | **p value*** | **p value**** | **p value***** | | **Experiments** |
| --- | --- | --- | --- | --- | --- | --- | --- | --- | --- | --- | --- | --- | --- | --- | --- |
| N2 |  |  | 191 | 24.5 | |  | |  |  | 24 |  |  |  | 3 | |
| N2 |  | 0x Peptone | 228 | 26.0 | |  | |  | 5.9 | 25 |  |  | 0.012 |  |  |
| N2 | BD |  | 178 | 31.5 | |  | | 28.4 |  | 32 |  | <.001 |  |  |  |
| N2 | BD | 0x Peptone | 246 | 31.4 | |  | | 21.0 | -0.3 | 30 |  | <.001 | 0.791 |  |  |
| N2 |  |  | 285 | 23.8 | |  | |  |  | 23 |  |  |  | 4 | |
| N2 | 5x Peptone |  | 205 | 30.4 | |  | | 27.7 |  | 31 |  | <.001 |  |  |  |
| N2 |  |  | 646 | 26.1 | |  | |  |  | 26 |  |  |  | 13 | |
| N2 | 10x Peptone |  | 663 | 29.8 | |  | | 14.2 |  | 30 |  | <.001 |  |  |  |
| N2 |  |  | 95 | 27.4 | |  | |  |  | 28 |  |  |  | 3 | |
| N2 | 1% Sorbitol |  | 85 | 32.3 | |  | | 17.9 |  | 33 |  | <.001 |  |  |  |
| N2 | 2.5 % Sorbitol |  | 101 | 36.3 | |  | | 32.7 |  | 35 |  | <.001 |  |  |  |
| N2 | 12.5% Sorbitol |  | 103 | 31.8 | |  | | 16.0 |  | 33 |  | 0.004 |  |  |  |
| N2 |  |  | 182 | 29.8 | |  | |  |  | 30.5 |  |  |  | 4 | |
| N2 | 5% Sorbitol |  | 180 | 35.9 | |  | | 20.5 |  | 37 |  | <.001 |  |  |  |
| daf-16 |  |  | 231 | 23.9 | | -19.6 | |  |  | 24 | <.001 |  |  |  |  |
| daf-16 | 5% Sorbitol |  | 270 | 27.5 | | -23.4 | | 14.8 |  | 28 | <.001 | <.001 |  |  |  |
| N2 |  |  | 73 | 28.9 | |  | |  |  | 28 |  |  |  | 2 | |
| N2 | 5% Sorbitol |  | 88 | 34.4 | |  | | 19.1 |  | 35 |  | <.001 |  |  |  |
| daf-2 |  |  | 131 | 54.1 | | 87.5 | |  |  | 54 | <.001 |  |  |  |  |
| daf-2 | 5% Sorbitol |  | 87 | 76.9 | | 123.6 | | 42.0 |  | 80 | <.001 | <.001 |  |  |  |
| N2 |  |  | 55 | 24.3 | |  | |  |  | 24 |  |  |  | 2 | |
| N2 | 5% Sorbitol |  | 131 | 41.1 | |  | | 69.3 |  | 42 |  | <.001 |  |  |  |
| sir-2.1 |  |  | 99 | 25.2 | | 3.6 | |  |  | 25 | 0.545 |  |  |  |  |
| sir-2.1 | 5% Sorbitol |  | 136 | 40.9 | | -0.6 | | 62.4 |  | 40 | 0.711 | <.001 |  |  |  |
| N2 |  |  | 173 | 25.7 | |  | |  |  | 26 |  |  |  | 3 | |
| N2 | 5% Sorbitol |  | 148 | 29.7 | |  | | 15.4 |  | 31 |  | <.001 |  |  |  |
| aak-2 |  |  | 167 | 23.7 | | -7.8 | |  |  | 24 | <.001 |  |  |  |  |
| aak-2 | 5% Sorbitol |  | 181 | 27.3 | | -8.2 | | 14.8 |  | 28 | <.001 | <.001 |  |  |  |
| N2 |  |  | 108 | 24.0 | |  | |  |  | 24 |  |  |  | 2 | |
| N2 | 5% Sorbitol |  | 81 | 27.6 | |  | | 15.0 | 15.0 | 28 |  | <.001 |  |  |  |
| hif-1 |  |  | 110 | 26.7 | | 11.0 | |  |  | 26 | 0.002 |  |  |  |  |
| hif-1 | 5% Sorbitol |  | 103 | 33.7 | | 21.8 | | 26.2 | 26.2 | 35 | <.001 | <.001 |  |  |  |
| N2 |  |  | 108 | 23.9 | |  | |  |  | 24 |  |  |  | 3 | |
| N2 |  | 5% Sorbitol | 190 | 39.5 | |  | |  | 65.7 | 38 |  |  | <.001 |  |  |
| N2 | BD |  | 214 | 33.7 | |  | | 41.3 |  | 32 |  | <.001 |  |  |  |
| N2 | BD | 5% Sorbitol | 182 | 37.1 | |  | | -6.2 | 10.0 | 35 |  | 0.012 | 0.029 |  |  |
| N2 |  |  | 55 | 24.3 | |  | |  |  | 24 |  |  |  | 2 | |
| N2 | 5% Sorbitol |  | 131 | 41.1 | |  | | 69.3 |  | 42 |  | <.001 |  |  |  |
| eat-2 |  |  | 65 | 26.0 | | 7.2 | |  |  | 25 | 0.100 |  |  |  |  |
| eat-2 | 5% Sorbitol |  | 88 | 43.1 | | 4.8 | | 65.6 |  | 43 | 0.212 | <.001 |  |  |  |
| N2 |  |  | 38 | 30.1 | |  | |  |  | 28 |  |  |  | 1 | |
| N2 | 100 mM NaCl |  | 41 | 28.1 | |  | | -6.3 |  | 28 |  | 0.274 |  |  |  |
| N2 |  |  | 80 | 25.4 | |  | |  |  | 26 |  |  |  | 1 | |
| N2 | 150 mM NaCl |  | 62 | 20.7 | |  | | -18.2 |  | 19 |  | <.001 |  |  |  |
| N2 |  |  | 47 | 31.2 | |  | |  |  | 34 |  |  |  | 1 | |
| N2 | 250 mM NaCl |  | 51 | 25.9 | |  | | -17.1 |  | 28 |  | 0.002 |  |  |  |
| N2 |  |  | 95 | 27.4 | |  | |  |  | 28 |  |  |  | 3 | |
| N2 | 500 mM NaCl |  | 118 | 7.2 | |  | | -73.7 |  | 7 |  | <.001 |  |  |  |
| N2 |  |  | 81 | 28.5 | |  | |  |  | 28 |  |  |  | 3 | |
| N2 |  | 5% Sorbitol | 99 | 35.3 | |  | |  | 23.7 | 37 |  |  | <.001 |  |  |
| N2 | Live OP50 |  | 108 | 27.3 | |  | | -4.3 |  | 28 |  | 0.212 |  |  |  |
| N2 | Live OP50 | 5% Sorbitol | 111 | 33.2 | |  | | -6.1 | 21.4 | 35 |  | 0.008 | <.001 |  |  |
| gpdh-1;gpdh-2 |  |  | 327 | 22.5 | |  | |  |  | 22 |  |  |  | 3 | |
| gpdh-1;gpdh-2 | 5% Sorbitol |  | 328 | 22,3 | |  | | -0.88 |  | 23 |  |  |  |  |  |
| gpdh-1;gpdh-2 | 200mM NaCl |  | 327 | 21.8 | |  | | -3.11 |  | 21 |  |  |  |  |  |
| osm-7 |  |  | 227 | 34.8 | |  | |  |  | 33 |  |  |  | 3 | |
| osm-7 | 5% Sorbitol |  | 189 | 45.8 | |  | | 31.60 |  | 41 |  | < 0.01 |  |  |  |
| osm-5 |  |  | 297 | 36.8 | |  | |  |  | 37 |  |  |  | 3 | |
| osm-5 | 5% Sorbitol |  | 256 | 48.8 | |  | | 32.60 |  | 49 |  | < 0.01 |  |  |  |

**Table S2. Nematode strains used in the study.**

| Strain Name | Genotype | Source |
| --- | --- | --- |
| N2 | Wildtype | CGC |
| CF1038 | daf-16 (mu86) | CGC |
| CB1370 | daf-2 (e1370) | CGC |
| VC199 | sir-2.1 (ok434) | CGC |
| RB754 | aak-2 (ok524) | CGC |
| ZG31 | hif-1 (ia04) | CGC |
| DA1113 | eat-2 (ad1113) | CGC |
| CB5602 | vhl-1 (ok161) | CGC |
|  | DAF-16::GFP | W. Li |
|  | GPDH-1::GFP | K. Strange |
| PR813 | osm-5 (p813) | CGC |
| MT3564 | osm-7 (n1515) | CGC |
|  | gpdh-1(ok1558);gpdh-2(kb33) | Todd Lamitina |
